# New Appliances and Things Medical

**Published:** 1898-10-01

**Authors:** 


### NEW APPLIANCES AND THINGS MEDICAL.

[We shall be glad to receive, at our Office 28 & 29, Southampton Street, Strand, London, W.C., from the manufacturers, specimens of all new preparations and appliances which may be brought out from time to time.]

### IVORY SOAP.

(Proctor and Gamble, Cincinnati; 15, Philpot Lane, London.)

This is a soap well adapted for laundry and domestic purposes. For bath, toilet, and nursery use its low specific gravity, which enables it to float in water, renders it extremely valuable. It appears to be a soap of careful manufacture and of great purity, and we highly recommend it to the notice of our readers.

